# Walking against traffic and pedestrian injuries in the United Kingdom: new insights

**DOI:** 10.1186/s12889-023-17083-8

**Published:** 2023-11-09

**Authors:** Akhmad Fajri Widodo, Chenyi Chen, Cheng-Wei Chan, Wafaa Saleh, Bayu Satria Wiratama, Chih-Wei Pai

**Affiliations:** 1 Present Address: Graduate Institute of Injury Prevention and Control, College of Public Health, Taipei Medical University, Taipei City, 110 Taiwan; 2 Research Center of Brain and Consciousness, Shuang-Ho Hospital, Taipei Medical University, New Taipei City, Taiwan; 3 Graduate Institute of Mind, Brain and Consciousness, College of Humanities and Social Sciences, Taipei Medical University, Taipei City, Taiwan; 4 Psychiatric Research Center, Wan Fang Hospital, Taipei Medical University, Taipei, Taiwan; 5 Neuroscience Research Center, Taipei Medical University Hospital, Taipei, Taiwan; 6 Department of Emergency Medicine, New Taipei City Hospital, New Taipei City, Taiwan; 7 College of Medicine, Chang Gung University, Taoyuan City, Taiwan; 8 Department of Emergency Medicine, Chang Gung Memorial Hospital, Taoyuan City, Taiwan; 9 Transport Research Institute, Edinburgh Napier University, Edinburgh, Scotland; 10 Department of Epidemiology, Biostatistics, and Population Health, Faculty of Medicine, Public Health and Nursing, Universitas Gadjah Mada, Yogyakarta City, 55281 Indonesia

**Keywords:** Walking against traffic, Walking with traffic, Pedestrians, Fatalities, Joint Effects

## Abstract

**Background:**

Studies from Finland and Taiwan have shown that walking against traffic was beneficial for reducing pedestrian crashes and fatalities. This study examined whether such beneficial effects are consistent across various circumstances.

**Methods:**

This study aimed to investigate pedestrian fatalities in walking-against or with-traffic crashes by analysing the UK STATS19 crash data for the period between 1991 and 2020. We firstly employed Chi-square tests to examine risk factors for pedestrian injury severity. These variables were then incorporated into stepwise logistic regression models with multiple variables. We subsequently conducted joint effect analysis to investigate whether the beneficial effects of walking against traffic on injury severity vary across different situations.

**Results:**

Our data contained 44,488 pedestrian crashes, of which 16,889 and 27,599 involved pedestrians walking against and with traffic, respectively. Pedestrians involved in with-traffic crashes were more likely to sustain fatalities (adjusted odds ratio [AOR] = 1.542; confidence interval [CI] = 1.139–1.927) compared with those in walking against-traffic crashes. The detrimental effect of walking with traffic on fatalities appeared to be more pronounced in darkness-unlit conditions (AOR = 1.48; CI = 1.29–1.70), during midnight hours (00:00–06:59 am) (AOR = 1.60; CI = 1.37–1.87), in rural areas (AOR = 2.20; CI = 1.92–2.51), when pedestrians were elderly (≥ 65 years old) (AOR = 2.65, CI = 2.16–3.26), and when heavy goods vehicles were crash partners (AOR = 1.51, CI = 1.28–1.78).

**Conclusions:**

Walking against traffic was beneficial in reducing pedestrian fatalities compared with walking with traffic. Furthermore, such a beneficial effect was more pronounced in darkness-unlit conditions, at midnights (00:00–06:59 am), in rural areas, when pedestrians were elderly, and when heavy goods vehicles struck pedestrians.

## Background

Pedestrians are among the most vulnerable road users because of their limited resistance to biomechanical forces and lack of mass, speed, and protection [1]. Pedestrians face relatively high risks of injury and death in traffic crashes, and such crashes have resulted in hundreds of thousands of pedestrian deaths annually worldwide [2]. From 2009 to 2019, pedestrian fatalities rose by 51% in the world. Moreover, a total of 1,300,000 pedestrians are killed in motor vehicle crashes annually [3]. Statistics revealed that approximately 75,000 pedestrians were injured in motor vehicle crashes in 2019 [4], accounting for 17% of all crash fatalities in that year. According to the US National Highway Traffic Safety Administration, most pedestrian crashes occur at intersections [5, 6]. Although the number of crashes occurring at non-intersection locations such as road segments is not as high as that of crashes occurring at road intersections, injuries sustained by pedestrians involved in road-segment crashes tend to be more severe than those sustained by pedestrians involved in intersection crashes [7].

Contraflow cycling schemes have been applied to improve cyclist safety and reduce crash risks [8]. The primary benefit of contraflow cycling schemes is that motor vehicles and cyclists can maintain continuous eye contact and perform evasive maneuvers to avoid crashes [9]. Such schemes have been adopted for pedestrians who must walk along road segments. The Finnish Road Traffic Act [10, 11] stipulates that pedestrians walk against traffic, and this mandate has been in effect for decades. Additionally, authorities in other countries encourage pedestrians to walk facing oncoming traffic in the absence of a sidewalk, pedestrian lane, or path [12].

Evidence from research conducted in Finland demonstrates that walking against traffic was beneficial in reducing pedestrian crash risks [13]. Similarly, a study conducted in Taiwan reported that pedestrians who walked with traffic appeared to have more severe injuries than did those who walked against traffic [14]. A possible explanation for the benefits of walking against traffic is that pedestrians walking against traffic are more visible than those walking with traffic, particularly at night [15].

### Purpose

The aforementioned studies clearly demonstrated that walking against traffic is advantageous for reducing both crash risks and severity. According to our knowledge, relatively few studies have examined whether the beneficial effect of walking against traffic on injury severity also applies to a set of conditions such as lighting conditions, crash time, roadway characteristics (rural/urban areas), ages, and vehicle types. Accordingly, the primary objective of the current study was to fill this research gap by investigating whether the benefits of walking against traffic are consistent across various circumstances involving such factors.

## Methods

### Data source

This study collected data on vehicle crashes for the period between 1991 and 2020 from the STATS19 crash database, a national traffic database of the United Kingdom. The STATS19 database contains data on every accident that involves at least one vehicle, results in personal injuries, and is reported to the UK police within a 30-day timeframe after the incident [16]. The database was created in 1949 and is periodically updated. Its datasets include an accident report, a vehicle file, and a casualty file. The accident report documents the time and date of a crash and the corresponding weather, road, and lighting conditions; the vehicle file contains information regarding the vehicle and its driver; and the casualty file contains information about each injury. This database is publicly accessible on the Department of Transportation's website [16].

**Fig. 1:**
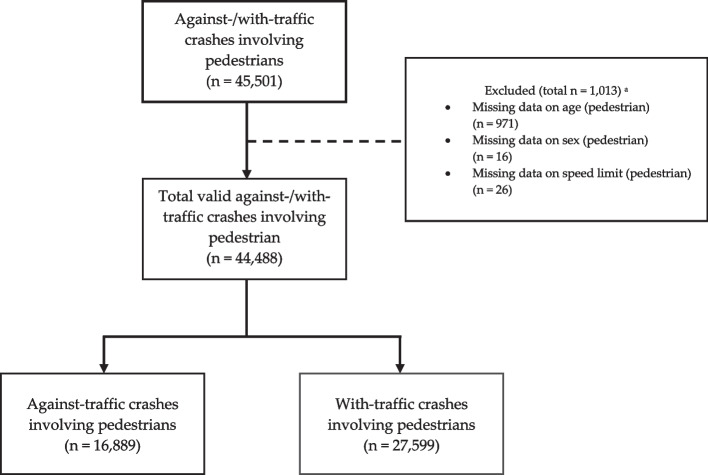
Sample selection flowchart. ^a^Exclusion criteria listed below are not exhaustive, total cases may exceed 1,013

Figure 1 illustrates a flowchart of the data selection process in this study. We excluded casualties with missing data on sex, age, and speed limit. We used a complete case analysis approach, as proposed by Kang [17]. Our collected contained 44,488 pedestrian crashes, of which 16,889 (38.0%) involved pedestrians walking against traffic and 27,559 (62.0%) involved pedestrians walking with traffic.

### Definitions of variables

We acquired the following demographic information on pedestrian casualties: sex, age, street lighting condition, crash time, speed limit, crash day and month, weather conditions, road surface conditions, crash partner (vehicle, sex, and age), and pedestrian walking direction (against traffic; with traffic). Age was categorized as follows: < 18, 18–40, 41–64, and ≥ 65 years. Moreover, the following variables were considered in the study: crash location (rural: roadways with speed limits of ≥ 51 km/h; urban: roadways with speed limits of ≤ 50 km/h), weather conditions (fine weather; adverse weather; or unknown), street lighting condition (daylight, lit streets in darkness, unlit streets in darkness, or unknown), road surface condition (dry; slippery), crash season (spring/summer: March–August; autumn/winter: September–February), crash day (weekday: Monday–Friday; weekend: Saturday–Sunday), and crash time (rush hour: 07:00–08:59 and 17:00–18:59.; non–rush hour: 09:00–16:59; evening: 19:00–23:59; and midnight/early morning: 00:00–06:59).

### Statistical analysis

We determined the distribution of pedestrian injury severity according to a set of independent variables (Table 1). Chi-square tests were then employed to examine the relationships between the independent variables and pedestrian injury severity and to identify variables significantly associated with the outcome variables (*p* < 0.05). These variables were then incorporated into logistic regression models with multiple variables. Subsequently, the odds of fatal and nonfatal injuries were estimated using stepwise logistic regression models after for specific variables were controlled for. In addition, joint effect analysis was used to examine the beneficial effects of walking against traffic on injury severity to different situations.

**Table 1:** Distribution of pedestrian injury severity according to independent variables

		Pedestrian injuries		
Variable	n (%)	Fatal	Non-fatal	χ^2^ test *p* value
		n (%)	n (%)	
Total	44,488	1,409 (3.2%)	43,079 (96.8%)	
Pedestrian movements				
Against traffic	16,889 (38.0%)	469 (2.8%)	16,420 (97.2%)	< 0.001
With traffic	27,599 (62.0%)	940 (3.4%)	26,659 (96.6%)	
Light conditions				
Daylight	28,521 (64.1%)	380 (1.3%)	28,141 (98.7%)	< 0.001
Darkness-lit	8,543 (19.2%)	212 (2.5%)	8,331 (97.5%)	
Darkness-unlit	6,634 (14.9%)	793 (12.0%)	5,841 (88.0%)	
Unknown	790 (1.8%)	24 (3.0%)	766 (97.0%)	
Crash time				
Midnight (0–6)	4,953 (11.1%)	448 (9.0%)	4,505 (91.0%)	< 0.001
Rush hours (7–8/17–18)	9,057 (20.4%)	200 (2.2%)	8,857 (97.8%)	
Non-rush hours (9–16)	20,933 (47.1%)	278 (1.3%)	20,655 (98.7%)	
Evening (19–23)	9,545 (21.5%)	483 (5.1%)	9,062 (94.9%)	
Speed limit				
Rural (≥ 40 mph)	11,824 (26.6%)	1,058 (8.9%)	10,766 (91.1%)	< 0.001
Urban (20–30 mph)	32,664 (73.4%)	351 (1.1%)	32,313 (98.9%)	
Crash day				
Weekend	12,610 (28.3%)	595 (4.7%)	12,015 (95.3%)	< 0.001
Weekday	31,878 (71.7%)	814 (2.6%)	31,064 (97.4%)	
Crash month				
Spring/summer	21,079 (47.4%)	584 (2.8%)	20,495 (97.2%)	< 0.001
Autumn/winter	23,409 (52.6%)	825 (3.5%)	22,584 (96.5%)	
Weather conditions				
Fine	36,641 (82.4%)	1,110 (3.0%)	35,531 (97.0%)	< 0.001
Adverse	6,944 (15.6%)	288 (4.1%)	6,656 (95.9%)	
Unknown	903 (2.0%)	11 (1.2%)	892 (98.8%)	
Road surface conditions				
Dry	31,944 (71.8%)	845 (2.6%)	31,099 (97.4%)	< 0.001
Slippery	12,465 (28.0%)	564 (4.5%)	11,901 (95.5%)	
Unknown	79 (0.2%)	0 (0.0%)	79 (100.0%)	
Pedestrian’s age				
≤ 18	10,643 (23.9%)	143 (1.3%)	10,500 (98.7%)	< 0.001
19–40	17,857 (40.1%)	632 (3.5%)	17,225 (96.5%)	
41–64	11,158 (25.1%)	417 (3.7%)	10,741 (96.3%)	
≥ 65	4,830 (10.9%)	217 (4.5%)	4,613 (95.5%)	
Pedestrian’s sex				
Male	27,670 (62.2%)	1,101 (4.0%)	26,569 (96.0%)	< 0.001
Female	16,818 (37.8%)	308 (1.8%)	16,510 (98.2%)	
Crash partner				
Pedal	683 (1.5%)	9 (1.3%)	674 (98.7%)	< 0.001
Motorcycle	1,248 (2.8%)	13 (1.0%)	1,235 (99.0%)	
Car/Taxi/Private hire car	32,361 (72.7%)	1,000 (3.1%)	31,361 (96.9%)	
Minibus/bus or coach	3,175 (7.1%)	55 (1.7%)	3,120 (98.3%)	
Heavy goods vehicles	6,882 (15.5%)	331 (4.8%)	6,551 (95.2%)	
Unknown	139 (0.3%)	1 (0.7%)	138 (99.3%)	
Crash partner’s age				
≤ 18	2,201 (4.9%)	84 (3.8%)	2,117 (96.2%)	< 0.001
19–40	16,603 (37.3%)	708 (4.3%)	15,895 (95.7%)	
41–64	10,538 (23.7%)	484 (4.6%)	10,054 (95.4%)	
≥ 65	3,007 (6.8%)	77 (2.6%)	2,930 (97.4%)	
Unknown	12,139 (27.3%)	56 (0.5%)	12,083 (99.5%)	
Crash partner’s sex				
Male	26,781 (60.2%)	1,174 (4.4%)	25,607 (95.6%)	< 0.001
Female	7,415 (16.7%)	187 (2.5%)	7,228 (97.5%)	
Unknown	10,292 (23.1%)	48 (0.5%)	10,244 (99.5%)	

## Results

As presented in Table 1, of the 44,488 pedestrian casualties, 1,409 were fatal (3.2%) and 43,079 (96.8%) were not. As many as 17,854 (40.1%) casualties were aged 19–40 years; although only 10.9% of the casualties were elderly pedestrians, their fatality rate was the highest among the age groups (4.5%). Regarding pedestrians’ movements, the number of fatal injuries was higher among pedestrians walking with traffic (27,559; 62.0%) than it was among those walking against traffic (16,889; 38.0%). Most of the pedestrian crashes occurred in daylight (28,521; 64.1%), during non–rush hour periods (09:00–16:59; 20,933; 47.1%), in urban areas (32,664; 73.4%), on weekdays (31,878; 71.7%), in autumn/winter (23,409; 52.6%), in fine weather (36,641; 82.4%), and on dry road surfaces (31,944; 71.8%); most of the pedestrian crashes also involved male pedestrians (27,670; 62.2%), cars as crash partners (32,361; 72.7%), crash partner drivers aged 19–40 years (16,603; 37.3%), and male crash partner drivers (26,781; 60.2%).

Table 2 presents the estimation results obtained from logistic regression models. The estimated parameter for walking with traffic was significant, suggesting that pedestrians in with-traffic crashes were 1.542 times more likely (adjusted odds ratio [AOR] = 1.542; confidence interval [CI] = 1.139–1.927) to sustain fatal injuries compared with those in against-traffic crashes. Other risk factors for fatal injuries include darkness-unlit condition (AOR = 2.349; CI = 1.949–2.939), midnight (0–6) (AOR = 3.023; CI = 2.369–3.859), rural roadways (AOR = 4.808; CI = 4.126–5.604), weekend (AOR = 1.202; CI = 1.062–1.360), elderly (AOR = 5.220, CI = 4.143–6.577), slippery road surfaces (AOR = 1.17; CI = 1.02–1.35), heavy goods vehicles as crash partners (AOR = 4.385, CI = 2.458–7.823) and male crash partner (AOR = 1.464; CI = 1.239–1.731).

**Table 2:** Multivariate logistic regression analysis results

Variable	AOR	95% CI	*p* value
Pedestrian movements			
Against traffic	Ref		< 0.001
With traffic	1.542	1.139–1.927	
Light condition			
Daylight	Ref		
Darkness-lit	1.422	1.123–1.800	0.003
Darkness-unlit	2.394	1.949–2.939	< 0.001
Unknown	1.456	0.921–2.302	0.108
Crash time			
Midnight (0–6)	3.023	2.369–3.859	< 0.001
Rush hours (7–8/17–18)	1.100	0.889–1.361	0.381
Non-rush hours (9–16)	Ref		
Evening (19–23)	2.015	1.607–2.527	< 0.001
Speed limit			
Rural (≥ 40 mph)	4.808	4.126–5.604	< 0.001
Urban (20–30 mph)	Ref		
Crash day			
Weekend	1.202	1.062–1.360	0.004
Weekday	Ref		
Crash month			
Spring/summer	Ref		
Autumn/winter	0.985	0.869–1.116	0.812
Weather conditions			
Fine	Ref		
Adverse	0.820	0.695–0.968	0.019
Unknown	0.523	0.281–0.972	0.040
Road surface conditions			
Dry	Ref		
Slippery	1.176	1.020–1.356	0.026
Unknown	< 0.001	0.000	0.997
Pedestrian’s age			
≤ 18	Ref		
19–40	1.567	1.292–1.901	< 0.001
41–64	2.300	1.880–2.815	< 0.001
≥ 65	5.220	4.143–6.577	< 0.001
Pedestrian’s sex			
Male	1.204	1.047–1.384	0.009
Female	Ref		
Crash partner			
Pedal	1.132	0.471–2.719	0.782
Motorcycle	Ref		0.782
Car/Taxi/Private hire car	2.705	1.532–4.777	0.001
Minibus/bus or coach	2.332	1.240–4.389	0.009
Heavy goods vehicles	4.385	2.458–7.823	< 0.001
Unknown	2.336	0.292–18.686	0.424
Crash partner’s age			
≤ 18	1.735	1.242–2.424	0.001
19–40	1.455	1.131–1.872	0.003
41–64	1.367	1.056–1.769	0.018
≥ 65	Ref		
Unknown	0.394	0.255–0.608	< 0.001
Crash partner’s sex			
Male	1.464	1.239–1.731	< 0.001
Female	Ref		
Unknown	0.412	0.270–0.628	< 0.001

**Fig. 2:**
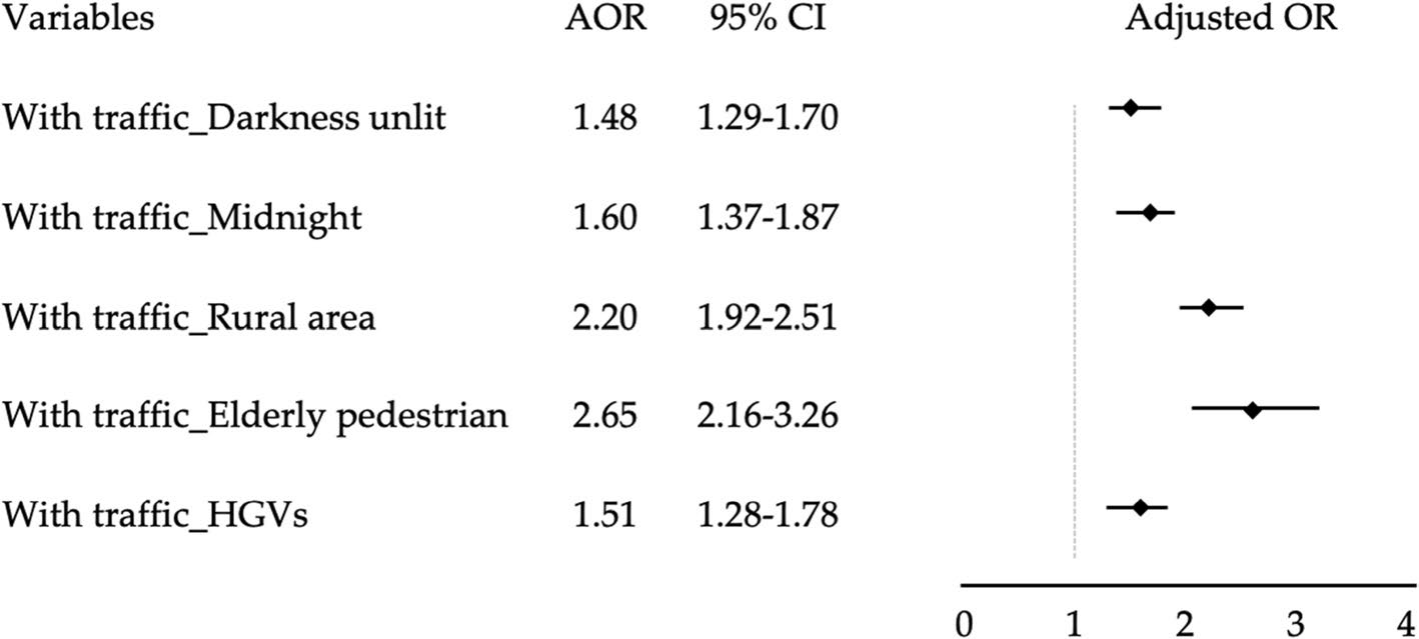
Joint effects of walking with traffic and other variables on pedestrian fatalities

Figure 2 displays a forest plot demonstrating the interaction effects of walking with traffic and other variables on fatalities. Walking with traffic may interact with other variables synergistically to intensify pedestrian injury severity. For example, pedestrian injury severity may be intensified through the synergistic interaction of walking with traffic with elderly pedestrians (AOR = 2.65, CI = 2.16–3.26), rural roadways (AOR = 2.20, CI = 1.92–2.51), heavy goods vehicles as crash partners (AOR = 1.51, CI = 1.28–1.78), unlit streets in darkness (AOR = 1.48, CI = 1.29–1.70), and midnight crashes (AOR = 1.60, CI = 1.37–1.87).

## Discussion

We analyzed accident data collected from the STATS19 database and determined that walking against traffic was more beneficial in reducing pedestrian fatalities during crashes than did walking with traffic. Our findings are in line with those of studies conducted in Finland and Taiwan [13, 14]. This study could be applied to these countries such as Japan and Spain where traffic compositions and urban design layout are similar to those in United Kingdom. These findings imply that mandating that pedestrians walk against traffic may not be practical in the United Kingdom; nevertheless, pedestrians should be encouraged to walk against traffic, especially on road sections without sidewalks, as suggested by Pai [14].

Our study contributes to research on pedestrian safety by demonstrating that the detrimental effect of walking with traffic on pedestrian fatalities was more pronounced under the following circumstances: in darkness-unlit condition, during midnight (00:00–06:59 am) hours, in rural areas, when the pedestrians were elderly (≥ 65 years old), when heavy goods vehicles were crash partners. Our findings support the notion that the benefits of walking with traffic are consistent across various circumstances, particularly in crashes involving elderly pedestrians.

Our study made several findings regarding the interaction effects of walking with traffic and other variables on fatalities. First, pedestrian crashes and injury severity could be increased by the interaction of walking with traffic with unlit streets in darkness, rural roadways, and midnight crashes [18–20]. This finding indicates that walking against traffic in these circumstances could help reduce pedestrian injury severity, and this suggestion can contribute to pedestrian safety. Although a previous study [19] recommended that pedestrians wear fluorescent or reflective garments to enhance their conspicuity, walking against traffic is also an effective countermeasure in rural areas, during midnight hours, or in unlit darkness.

Second, walking with traffic could interact with age to increase pedestrian fatalities, particularly among elderly pedestrians. Research [21, 22] has pointed out that clothing with retroreflective materials can make pedestrians, particularly elderly pedestrians, more conspicuous. Additionally, we recommend that pedestrians, especially older pedestrians, should walk against traffic, which may enhance the effectiveness of their reflective clothing and increases their conspicuity, consequently reducing their fatality risk.

Third, the detrimental effect of walking with traffic on fatalities could be accentuated by heavy goods vehicles as crash partners. This is because blind spots close to heavy goods vehicles may be exacerbated when pedestrians walk with traffic, allowing fewer opportunities for evasive action [23, 24]. The combination of walking against traffic and improved pedestrian conspicuity through pedestrian detection systems could be an intervention focus to reduce both crashes and injury severity.

Our study inevitably has several limitations. First, geometric characteristics, such as the presence of sidewalks and vertical/horizontal road curvatures, are not readily available in the STATS19 database; these factors affect pedestrian conspicuity, crash risks, and fatalities. Second, other factors such as alcohol use and fatigue, which may be more prevalent during midnight hours, were not considered in this study; they should thus be investigated further in future studies. Finally, although police-reported crash data generally contain no information on mobile phone use either by vehicle drivers or pedestrians, the impact of this factor on pedestrian crashes and fatalities should be explored further.

## Conclusions

We determined that walking against traffic was beneficial in reducing pedestrian fatalities compared with walking with traffic. These benefits were particularly pronounced under circumstances involving unlit streets in darkness, midnight driving (00:00–06:59), rural roadways, elderly pedestrians, and heavy goods vehicles as crash partners.

## Data Availability

The current research used the U.K. STATS19 database, which contains data on all road traffic accidents in the United Kingdom. The data that support the findings of this study are openly available in https://www.data.gov.uk/dataset/cb7ae6f0-4be6-4935-9277-47e5ce24a11f/road-safety-data.

## Abbreviations

AOR: Adjusted odds ratio
CI: Confidence interval
SD: Standard deviation
